# Estragole Inhibits Growth and Aflatoxin Biosynthesis of *Aspergillus flavus* by Affecting Reactive Oxygen Species Homeostasis

**DOI:** 10.1128/spectrum.01348-23

**Published:** 2023-06-08

**Authors:** Liuke Liang, Wei Zhang, Jing Hao, Yanyu Wang, Shan Wei, Shuaibing Zhang, Yuansen Hu, Yangyong Lv

**Affiliations:** a College of Biological Engineering, Henan University of Technology, Zhengzhou, China; Yangzhou University

**Keywords:** estragole, *Aspergillus flavus*, aflatoxin biosynthesis, transcriptomics

## Abstract

A variety of essential oils and edible compounds have been widely recognized for their antifungal activity in recent years. In this study, we explored the antifungal activity of estragole from Pimenta racemosa against Aspergillus flavus and investigated the underlying mechanism of action. The results showed that estragole had significant antifungal activity against A. flavus, with a minimum inhibitory concentration of 0.5 μL/mL against spore germination. Additionally, estragole inhibited the biosynthesis of aflatoxin in a dose-dependent manner, and aflatoxin biosynthesis was significantly inhibited at 0.125 μL/mL. Pathogenicity assays showed that estragole had potential antifungal activity against A. flavus in peanut and corn grains by inhibiting conidia and aflatoxin production. Transcriptomic analysis showed that the differentially expressed genes (DEGs) were mainly related to oxidative stress, energy metabolism, and secondary metabolite synthesis following estragole treatment. Importantly, we experimentally verified reactive oxidative species accumulation following downregulation of antioxidant enzymes, including catalase, superoxide dismutase, and peroxidase. These results suggest that estragole inhibits the growth and aflatoxin biosynthesis of A. flavus by modulating intracellular redox homeostasis. These findings expand our knowledge on the antifungal activity and molecular mechanisms of estragole, and provide a basis for estragole as a potential agent against A. flavus contamination.

**IMPORTANCE** Aspergillus flavus contaminates crops and produces aflatoxins, carcinogenic secondary metabolites which pose a serious threat to agricultural production and animal and human health. Currently, control of A. flavus growth and mycotoxin contamination mainly relies on antimicrobial chemicals, agents with side effects such as toxic residues and the emergence of resistance. With their safety, environmental friendliness, and high efficiency, essential oils and edible compounds have become promising antifungal agents to control growth and mycotoxin biosynthesis in hazardous filamentous fungi. In this study, we explored the antifungal activity of estragole from *Pimenta racemosa* against A. flavus and investigated its underlying mechanism. The results demonstrated that estragole inhibits the growth and aflatoxin biosynthesis of A. flavus by modulating intracellular redox homeostasis.

## INTRODUCTION

Aspergillus flavus is a common pathogenic fungus, especially in oil-containing crops such as peanut, cottonseed, and corn, where it causes rot and mildew, resulting in serious crop yield losses (1, 2). In humans, A. flavus is the second leading cause of allergic, invasive, and colonizing fungal diseases, and its conidia can colonize the respiratory tract and cause invasive aspergillosis (IA) (3). A. flavus also produces the carcinogenic secondary metabolite aflatoxin, which is a severe threat to food safety and human health (4, 5). Aflatoxin can penetrate human skin and enter the circulatory system, interfering with nucleic acid synthesis in cell nuclei, disrupting cell metabolic processes, causing immunosuppression, and damaging DNA, resulting in irreparable damage (6). In the United States, aflatoxin contamination causes annual economic losses of nearly $1.68 billion in corn production and processing (7). Additionally, consumption of corn, peanuts, and cottonseeds contaminated with aflatoxin causes death in animals and humans (8). Related studies have confirmed that the high incidence of liver cancer and other cancers worldwide is closely related to A. flavus and aflatoxin contamination (9). Therefore, it is necessary to explore effective methods for managing A. flavus growth and mycotoxin contamination.

Currently, the control of A. flavus growth and mycotoxin contamination relies on aromatic hydrocarbons, benzimidazoles, biosynthesis inhibitors of sterol production, and other antimicrobial chemicals (10–12). The use of high concentrations of these chemicals is often required for their efficacy, but side effects such as carcinogenicity, teratogenicity, high levels of toxic residues, and the emergence of resistance have been reported (13, 14). Faced with these considerable disadvantages and threats, there is growing interest in finding safer, more effective natural substances such as essential oils, microbial volatile organic compounds, and medicinal and edible compounds to prevent A. flavus growth and aflatoxin contamination (15–17). In recent years, essential oils, including aldehydes, phenols, alcohols, and ketones, have been recognized as natural fungicides, effective biocontrol agents, and nontoxic biological preservatives (13, 18–20). Among these essential oils, estragole, a volatile terpenoid with a safe concentration range of <1.9242 mg/kg of body weight per day according to the FDA (https://www.fda.gov/search?s=estragole), is a natural ingredient in various herbs and spices, and its essential oils are often used in food additives, soaps, and detergents (21, 22). It has been reported that extracts of Pimenta racemosa containing estragole possess significant antibacterial activity that can inhibit Pseudomonas syringa and control bacterial canker disease in kiwifruit (23). Furthermore, *in vitro* antimicrobial activity against Microsporum canis (24) and food spoilage fungus (25) has been demonstrated. However, there have been no reports about the inhibitory effects of estragole on A. flavus growth and contamination, and the possible inhibitory mechanism remains to be explored.

In this work, we determined the minimum inhibitory concentration (MIC) of estragole on the germination of A. flavus. Additionally, we also characterized A. flavus growth, aflatoxin production, hyphae dry weight, and growth inhibition of A. flavus infecting peanut and corn. Furthermore, RNA sequencing (RNA-seq) transcriptional analysis was performed to unveil the underlying mechanism of estragole in inhibiting growth and aflatoxin synthesis in A. flavus. Differentially expressed genes (DEGs) were mainly enriched in oxidative stress, energy metabolism, and the biosynthesis of secondary metabolites, including aflatoxin, gliotoxin, and ustiloxin B. Reactive oxygen species (ROS) were measured, as were the activities of the antioxidant-related enzymes catalase (CAT), peroxidase (POD), and superoxide dismutase (SOD). Our results expand knowledge of aflatoxin biosynthesis and may assist in the development of effective control measures for aflatoxin contamination.

## RESULTS

### Inhibitory effects of estragole on *A. flavus* spore germination.

**FIG 1:**
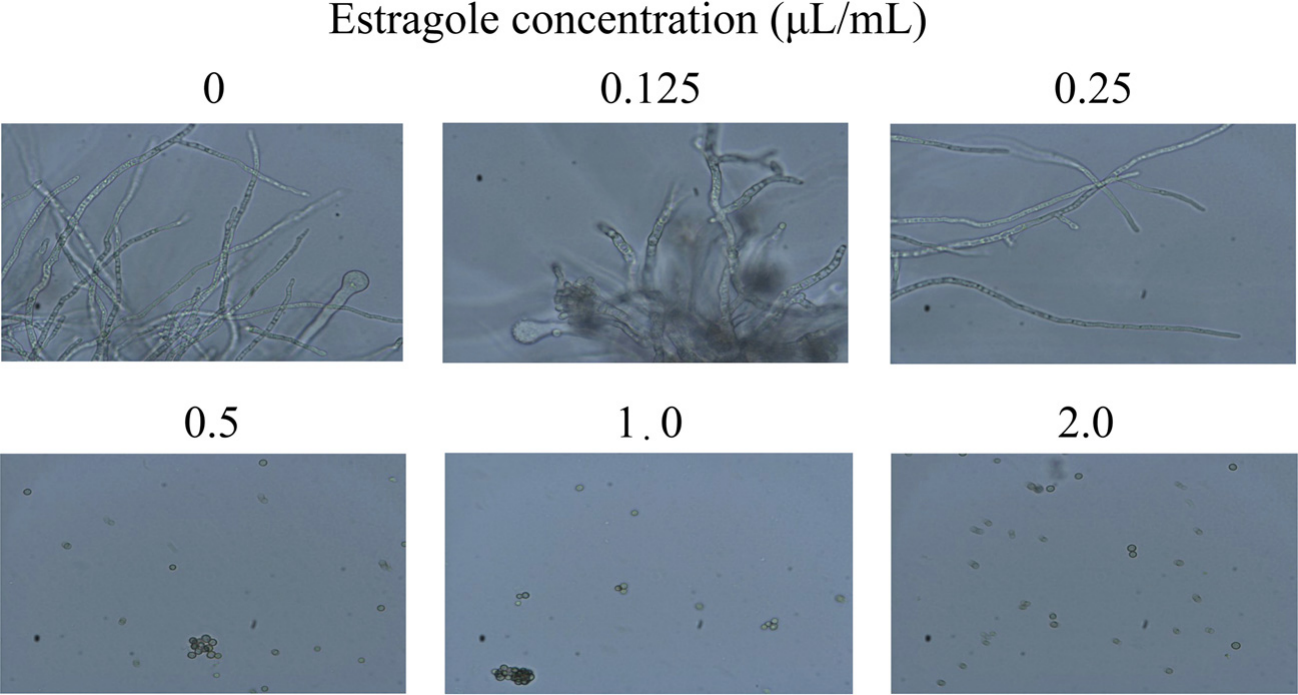
Effect of estragole on spore germination of Aspergillus flavus. Different concentrations of estragole were added into potato dextrose broth (PDB) medium and cultured at 30°C for 24 h and the germination of A. flavus spores was observed under a microscope (×400).

To determine the MIC of estragole on A. flavus spore germination, spores exposed to different concentrations of estragole were cultured in potato dextrose broth (PDB) medium for 24 h. Spore germination was observed under a microscope, and the results indicated that all untreated spores germinated. Low concentrations of estragole (0.125 and 0.25 μL/mL) did not inhibit A. flavus growth, while spores had not germinated at 24 h after treatment with estragole at concentrations of 0.5, 1.0, and 2.0 μL/mL (Fig. 1). Therefore, we concluded that the MIC of estragole for inhibiting A. flavus spore germination was 0.5 μL/mL.

To further investigate the killing of A. flavus spores by estragole, we performed propidium iodide (PI) and Hoechst staining experiments. The results showed that the fluorescence distribution of spores did not shift after treatment with different estragole concentrations, suggesting that exogenous estragole did not alter spore death in a dose- and time-dependent manner (Fig. S1 in the supplemental material). Therefore, to explore the inhibitory effect of estragole on cell growth, development, and aflatoxin biosynthesis in A. flavus more comprehensively, we selected a 0.125 μL/mL concentration of estragole to treat A. flavus spores for transcriptomic analysis according to a previous study (26).

### Effects of estragole on the growth and aflatoxin biosynthesis of *A. flavus*.

**FIG 2:**
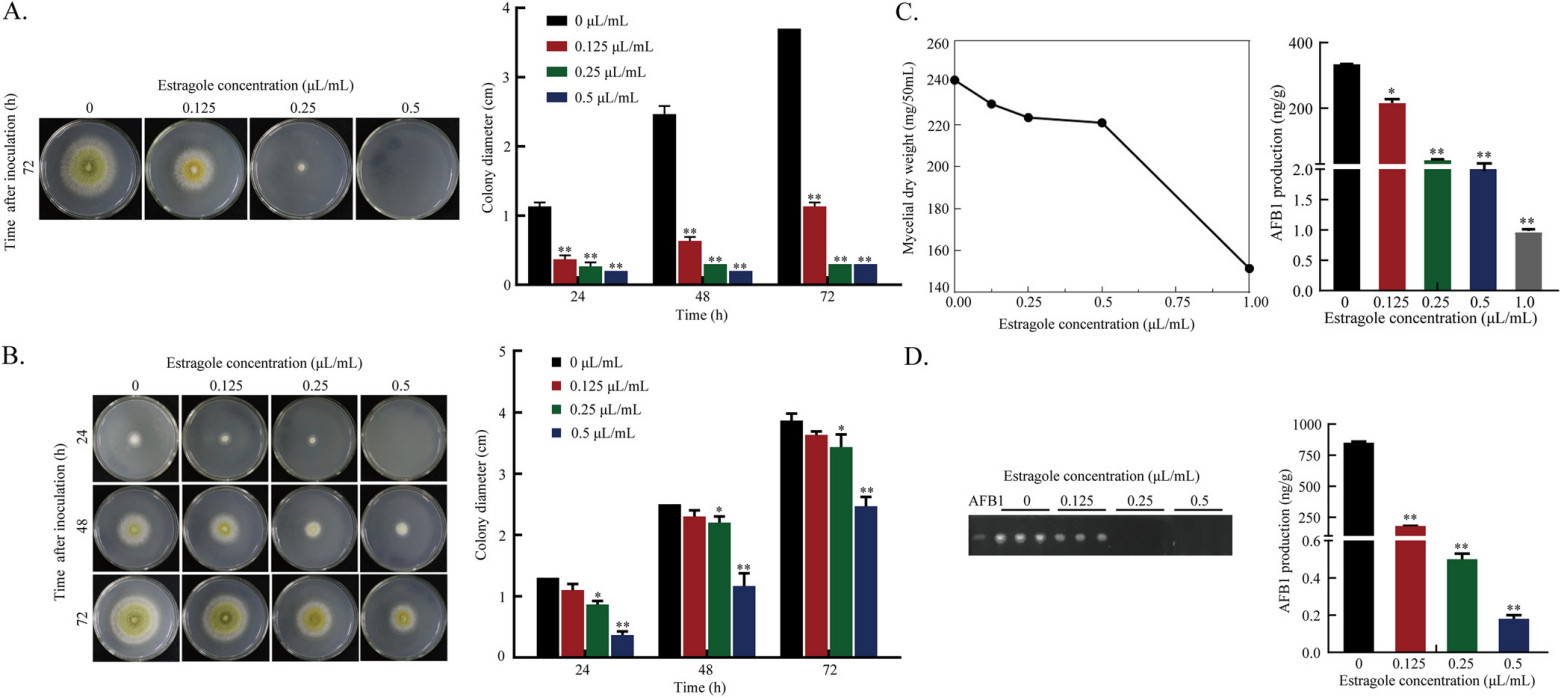
Effect of estragole on growth and toxicity of A. flavus. (A) Effects of different concentrations of estragole on the growth of A. flavus under fumigation. (B) Estragole was added into PDA medium, inoculated with A. flavus spore suspensions of 10^6^ spores/mL, and cultured at 30°C for 72 h. (C) The dry weight of A. flavus mycelium and AFB_1_ production was determined after estragole treatment under liquid culture conditions; (D) TLC and HPLC analysis of AFB_1_ production from panel B at 30°C for 72 h. Values are given as the mean (*n* = 3) ± standard deviation (*, *P* ≤ 0.05; **, *P* ≤ 0.001).

The inhibitory effects of estragole on the growth of A. flavus were characterized by gas-phase fumigation and solid culture. Under these two treatment conditions, estragole had a strong inhibitory effect on A. flavus growth. With increasing estragole concentrations, the growth diameter of A. flavus colonies decreased significantly. Under fumigation, the morphology of colonies treated with 0.25 μL/mL estragole was quite different from that of the untreated controls. Colonies in the control group showed loose green villi and produced conidiospores, while colonies in the treatment group remained pure white, and A. flavus growth was completely inhibited by 0.5 μL/mL estragole (Fig. 2A and B). Additionally, the mycelia dry weight was measured after 48 h; the results showed a gradual decrease with increasing estragole concentrations, and 1 μL/mL estragole significantly inhibited mycelial growth (Fig. 2C).

To characterize aflatoxin biosynthesis in A. flavus, aflatoxin and sterigmatocystin levels were measured at 72 h using thin-layer chromatography (TLC) and high-performance liquid chromatography (HPLC). Biosynthesis of aflatoxin B_1_ (AFB_1_) was dependent on estragole concentration; with increasing estragole concentrations, AFB_1_ biosynthesis gradually decreased, and at a concentration of 0.25 μL/mL, AFB_1_ synthesis was significantly inhibited (Fig. 2D). However, sterigmatocystin production by A. flavus was undetectable (Fig. S2). These results suggested that estragole inhibited the growth of A. flavus and the biosynthesis of AFB_1_.

### Effects of estragole on growth and aflatoxin biosynthesis in *A. flavus* on peanut and corn grains.

**FIG 3:**
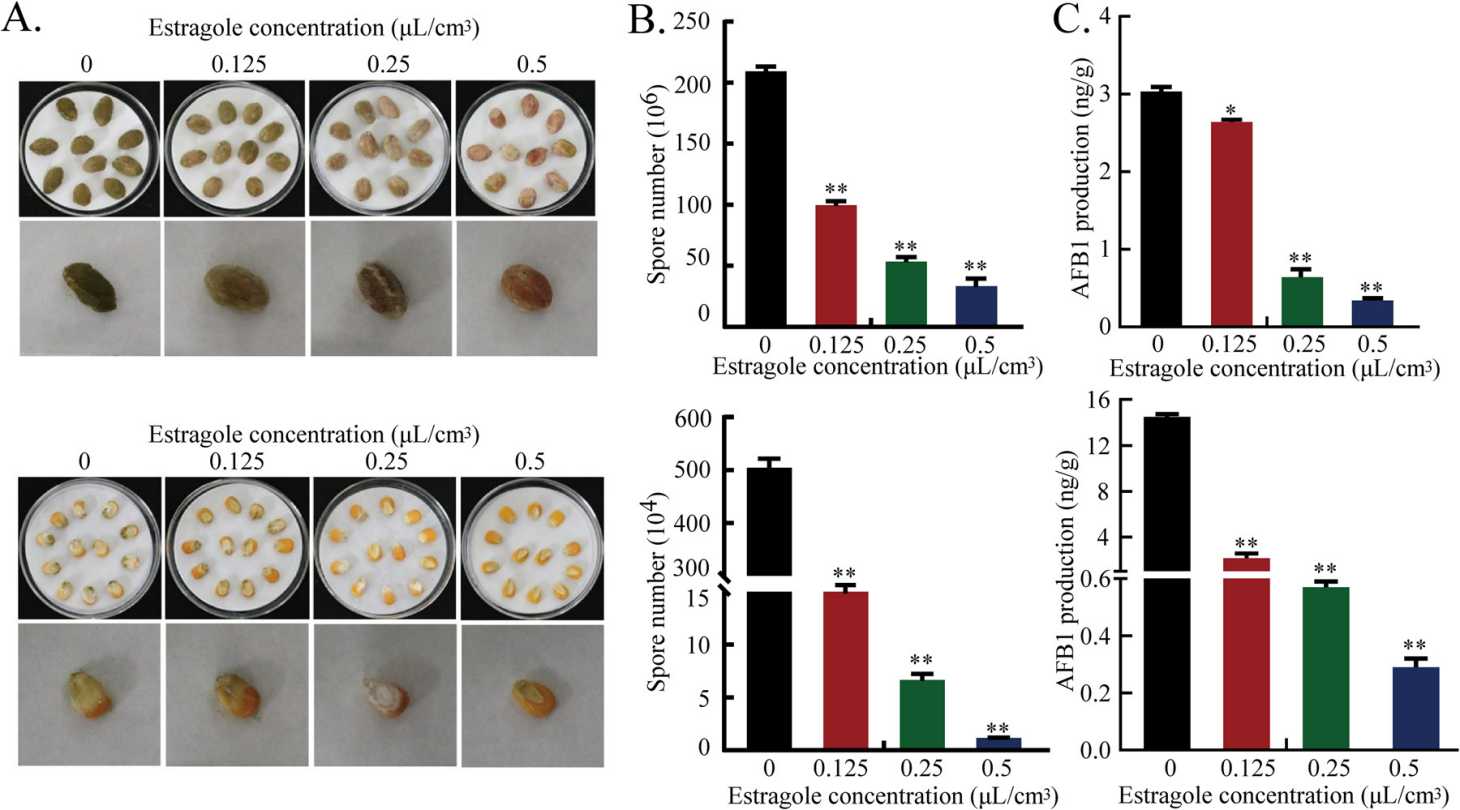
Effect of estragole on the growth and aflatoxin biosynthesis of A. flavus in crops. (A) Colonization of A. flavus on peanut and corn treated with different concentrations of estragole: A. flavus spore suspensions of 10^6^ spores/mL were inoculated on peanut and corn, estragole at concentrations of 0.125, 0.25, and 0.5 μL/cm^3^ was added to the lid according to the volume of the dish for fumigation, and the culture was sealed for 5 d. (B) The number of conidia on infected peanut and corn grains was determined. (C) HPLC analysis of AFB_1_ production on infected peanut and corn grains. Estragole concentrations are given in μL/cm^3^, meaning the amount of estragole (μL) per volume of petri dish (cm^3^). Values are given as the mean (*n *= 3) ± standard deviation (*, *P* ≤ 0.05; **, *P* ≤ 0.001).

To assess the inhibitory effects of estragole on growth and aflatoxin biosynthesis in A. flavus on peanut and corn seeds, we inoculated spore suspensions and counted the spores on the seed surface after culture. The results indicated that estragole strongly inhibited the development of A. flavus cells after 5 days of incubation. Compared with that in the controls, 0.5 μL/mL estragole treatment inhibited spore production on peanut and corn grains by 84.08% and 99.77%, respectively (Fig. 3A and B). Additionally, the production of AFB_1_ on infected peanut and corn grains decreased significantly after estragole fumigation (Fig. 3C).

### Overview of transcriptional analysis.

**FIG 4:**
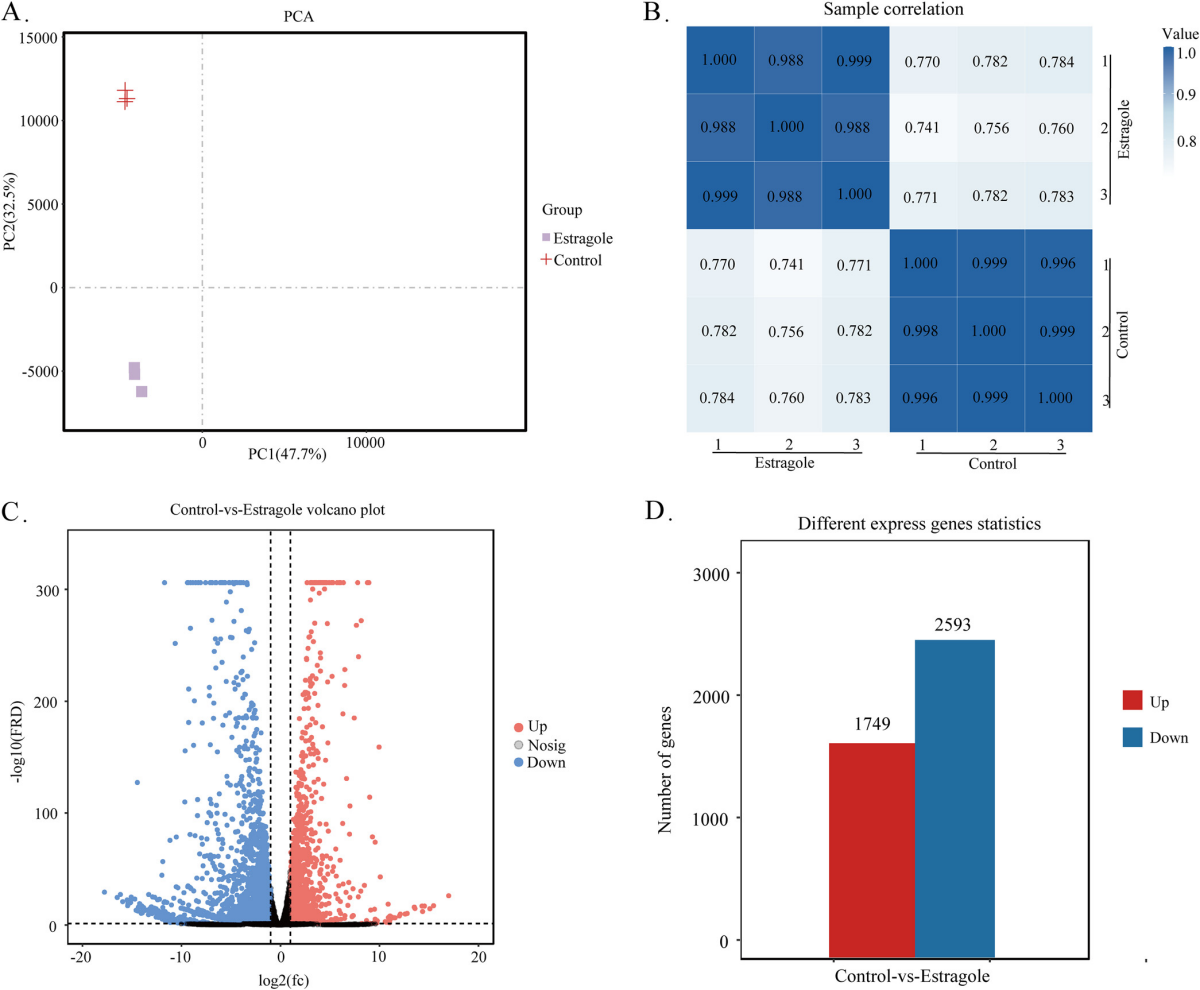
(A) PCA analysis. (B) Pearson correlation coefficient. (C) Volcano map of DEGs. (D) Number of DEGs.

To evaluate the quality of samples for RNA-seq analysis, correlations between samples were calculated. Principal-component analysis (PCA) showed that treated and untreated groups were similar, and groups were well discriminated (Fig. 4A). The Pearson correlation coefficient was >0.996 (Fig. 4B). These results indicated that the data were suitable for subsequent analysis.

Based on bioinformatic analysis, DEGs were defined with a false discovery rate (FDR) of <0.05 and a |log_2_(FC)| of >1 as the cutoff criteria, and the gene distribution and expression fold change (FC) were visualized using a volcano plot (Fig. 4C). The results showed that 4,342 genes were significantly differentially expressed, of which 1,749 (40.28%) were upregulated and 2,593 (59.72%) were downregulated (Fig. 4D).

### Functional enrichment analysis of DEGs.

**FIG 5:**
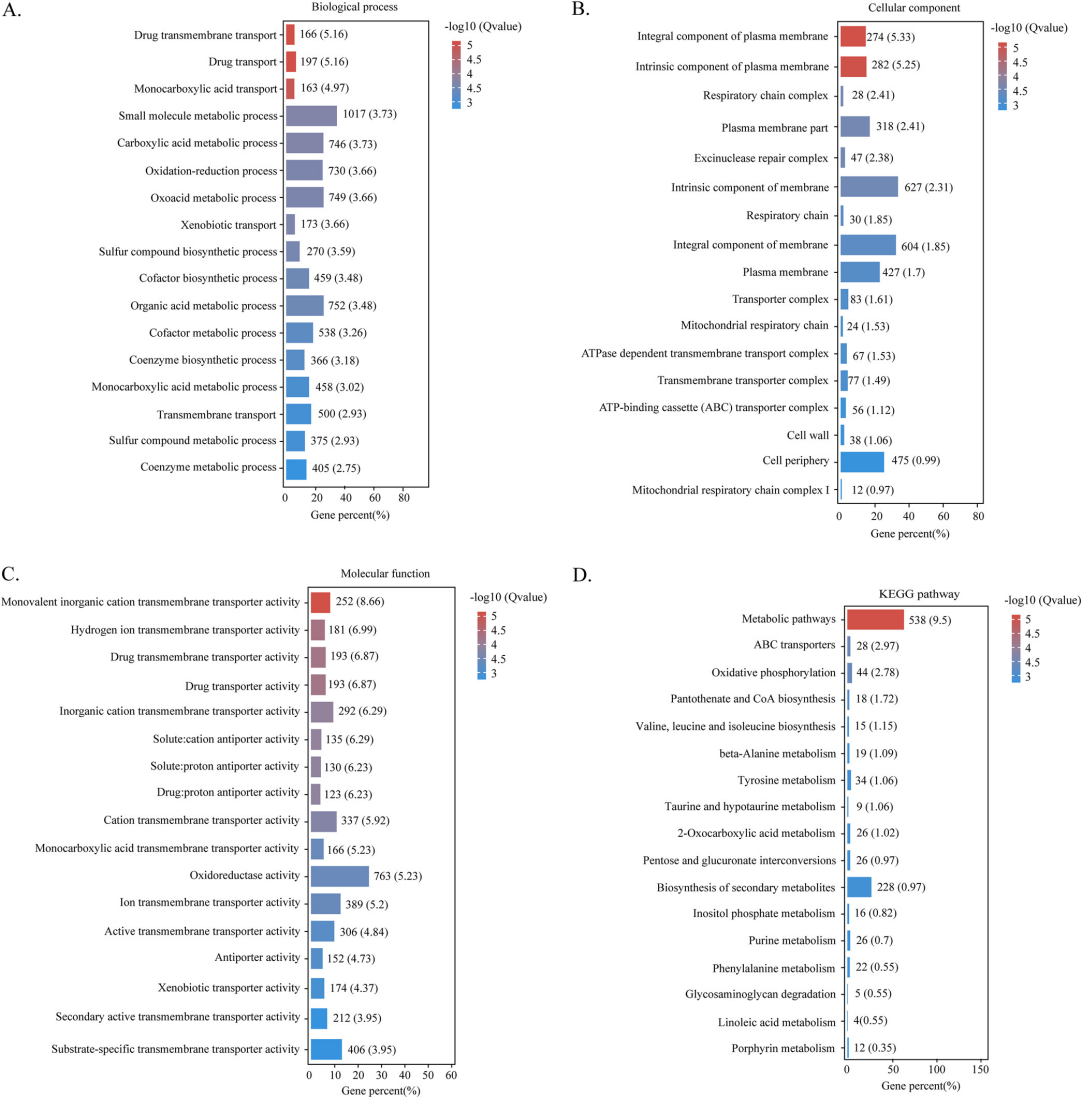
Functional enrichment analysis of DEGs in terms of (A) biological processes, (B) cellular components, (C) molecular function, and (D) KEGG pathway.

To further explore the biological functions of DEGs, we performed functional enrichment analysis. Gene ontology (GO) enrichment analysis showed that DEGs were linked to metabolic process, oxidation-reduction process, transport, plasma membrane, oxidoreductase activity, and transporter activity categories (Fig. 5A to C). Additionally, Kyoto Encyclopedia of Genes and Genomes (KEGG) enrichment analysis showed that most DEGs were associated with metabolic pathways, biosynthesis of secondary metabolites, and oxidative phosphorylation pathways (Fig. 5D). These results indicated that estragole might play a regulatory role in AFB_1_ biosynthesis by influencing secondary metabolic pathways via redox and energy homeostasis, including ROS content and the activities of antioxidant enzymes such as CAT, POD, and SOD.

### Categorization of DEGs.

To further unveil the inhibitory effects of estragole on the growth and aflatoxin biosynthesis of A. flavus, representative DEGs were categorized into six groups: oxidative stress, energy metabolism, secondary metabolism, cell growth and development, cell wall, and cell membrane (Table 1).

**TABLE 1:** Classification of representative DEGs in the estragole treatment group versus the control group

Gene category	Log_2_(FC)^*a*^	Name	Description
Oxidative stress			
AFLA_096210	−7.91	*ctl-2*	Catalase
AFLA_034380	−4.89	*cta1*	Catalase
AFLA_004510	−4.51	*pod*	Peroxidase
AFLA_027780	−2.04	*GSTU21*	Glutathione-*S*-transferase omega
AFLA_122020	−4.72	*2ODD21*	Oxidoreductase
AFLA_010030	−7.14	*hxnY*	Oxidoreductase
AFLA_059950	−6.37	*yanF*	Oxidoreductase
AFLA_092090	−3.11	*yhdF*	Oxidoreductase
Energy metabolism			
AFLA_015810	−2.77	*R3*	Citrate synthase
MSTRG.4380	−1.14	*ACL1*	Citrate synthase-like protein
AFLA_107660	−5.80	*tca-9*	Succinyl-CoA synthetase beta subunit
AFLA_137390	−1.23	*mdh*	Malate/l-lactate dehydrogenase
AFLA_015860	−2.04	*acoC*	Aconitase
AFLA_084130	−2.18	*kgd1*	Alpha-ketoglutarate dehydrogenase complex Subunit Kgd1
AFLA_035290	−1.41	*PDA1*	Pyruvate dehydrogenase
AFLA_073260	−2.03	*hxkA*	Hexokinase
AFLA_046760	−1.76	*gld1*	Glycerol 3-phosphate dehydrogenase
AFLA_137160	−1.99	*mug14*	Aldolase
AFLA_067640	−1.14	*NDH2*	NADH-dehydrogenase
Secondary metabolism			
AFLA_105170	−7.58	*aflO*	*O*-methyltransferase
AFLA_138050	−3.83	*aflQ*	Flavonoid 3-hydroxylase
AFLA_065760	−7.07	*aflU*	Cytochrome P450
AFLA_139420	−5.59	*aflT*	AflT/aflT/transmembrane protein
AFLA_139370	−1.66	*aflB*	AflB/fas-1/fatty acid synthase beta subunit
AFLA_139410	−1.53	*aflC*	AflC/pksA/pksL1/polyketide synthase
AFLA_005460	−8.62	*dtpC*	Cytochrome P450
AFLA_125750	−6.26	*CYP52A2*	Cytochrome P450 oxidoreductase/alkane
AFLA_094960	−14.01	*ustC*	Cytochrome P450
AFLA_094990	−13.66	*ustYa*	Ustiloxin B biosynthesis protein Ya
AFLA_095010	−9.15	*ustP*	Ustiloxin B biosynthesis protein P
AFLA_094940	−9.05	*ustO*	Ustiloxin B biosynthesis protein O
AFLA_095030	-4.33	*ustH*	Gamma glutamyl transpeptidase
AFLA_095040	−10.53	*ustD*	NRPS-like enzyme
AFLA_127090	−1.95	*pksCT*	Polyketide synthase
AFLA_060010	−15.13	*nscA*	PKS-like enzyme
AFLA_139490	−4.17	*cpaA*	Hybrid PKS/NRPS enzyme
AFLA_064560	−5.27	*aclP*	Nonribosomal peptide synthase GliP-like
AFLA_010620	−1.23	*NRPS4*	Nonribosomal siderophore peptide synthase Sid2
Cell growth and development			
AFLA_052030	−3.99	*wetA*	Developmental regulatory protein WetA
AFLA_029620	−6.92	*abaA*	Transcription factor AbaA
AFLA_026900	−2.79	*vosA*	Developmental regulator VosA
AFLA_046990	−2.08	*stuA*	APSES transcription factor StuA conidial
AFLA_092390	−2.07	*boi2*	Polarized growth protein (Boi2)
AFLA_133610	−1.14	*kog1*	TORC1 growth control complex subunit Kog1
Cell wall and cell membrane			
AFLA_134100	−7.18	*ags1*	Alpha-1,3-glucan synthase Ags2
AFLA_052780	−6.58	*Scw4*	Cell wall glucanase (Scw4)
AFLA_095960	−1.49	*chsD*	Chitin synthase D
AFLA_136030	−1.00	*chsE*	Chitin synthase ChsE
AFLA_102010	−9.10	*chit1*	Class V chitinase
AFLA_081820	−8.17	*dcw1*	Glycosyl hydrolase
AFLA_018750	−3.58	*gpi13*	Phosphatidylinositol glycan
AFLA_118200	−9.38	*dhcr7*	C-14 sterol reductase

a FC, fold change.

Antioxidant-related enzymes such as CAT (*ctl-2*, *cta1*), POD (*pod*), and oxidoreductase-related genes (*GSTU21*, *2ODD21*, *hxnY*, *yanF*, *yhdF*) were significantly downregulated. Similarly, genes encoding enzymes involved in energy metabolism pathways, such as *R3*, *ACL1*, *tca-9*, *mdh*, *acoC*, *kgd1*, *PDA1*, *hxkA*, *gld1*, and *mug14*, were significantly downregulated.

Estragole treatment affected the expression of genes involved in secondary metabolism. The results demonstrated that the AFB_1_ biosynthetic pathway genes *aflO*, *aflQ*, *aflU*, *aflT*, *aflB*, and *aflC*, as well as genes encoding cytochrome P450 (*dtpC*, *CYP52A2*), were downregulated after estragole treatment. Genes associated with the synthesis of secondary metabolites identified in other fungi, including polyketides (*pksCT*, *nscA*), nonribosomal polypeptides (*aclP*, *NRPS4*), and ustiloxin B synthesis genes (*ustC*, *ustYa*, *ustP*, *ustO*, *ustH*, *ustD*), were also downregulated. Additionally, estragole inhibited the expression of the spore-related genes *wetA*, *abaA*, and *stuA*, and the cell wall and membrane formation genes *ags1*, *Scw4*, *chsD*, *chsE*, *chit1*, *dcw1*, *gpi13*, and *dhcr7*.

### Effects of estragole on ROS, H_2_O_2_, O_2_^−^ accumulation, and CAT, POD, and SOD enzyme activities.

**FIG 6:**
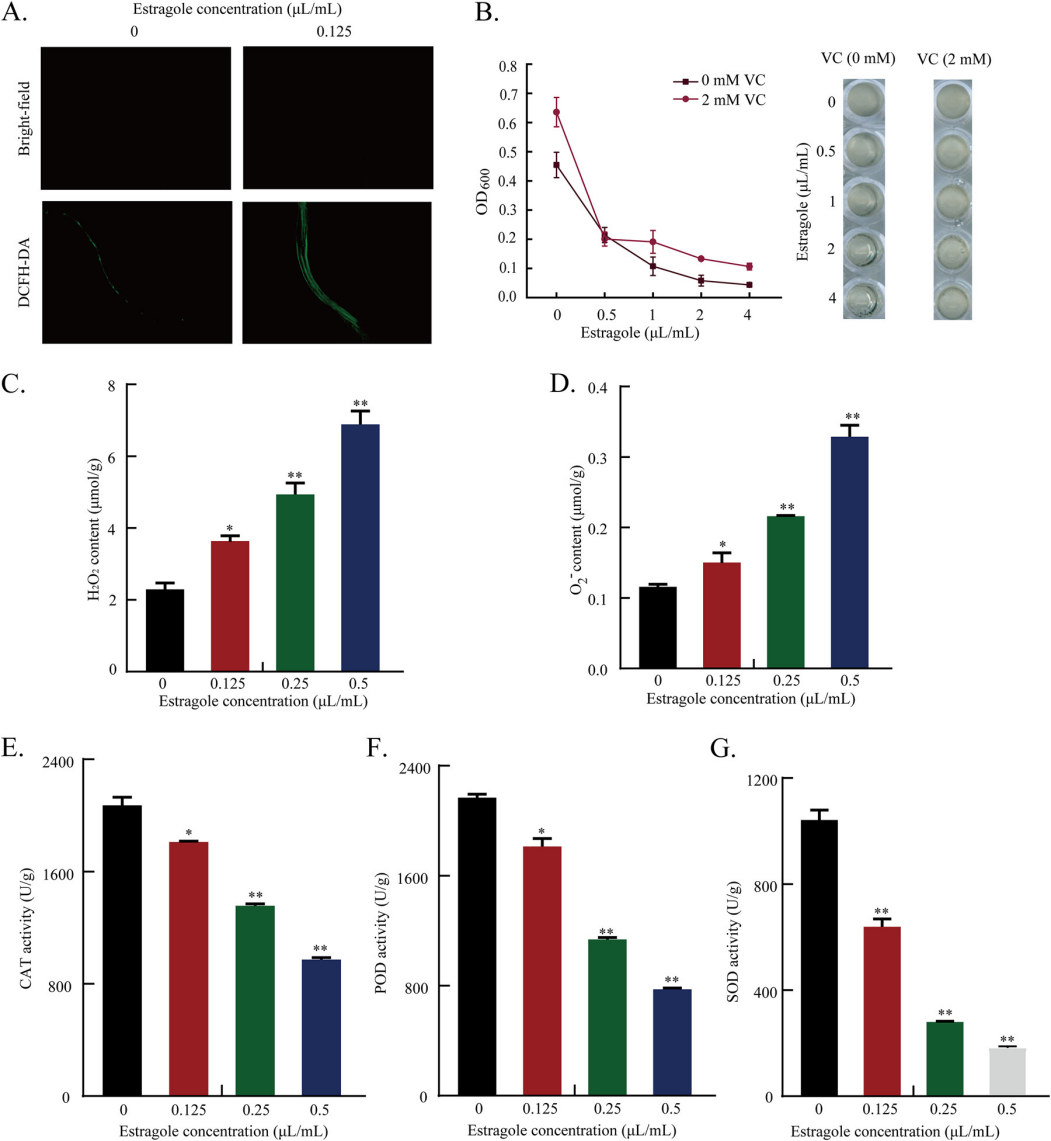
Effect of estragole on accumulation of ROS. (A) DCFH-DA dyeing. (B) The inhibitory effect of estragole on A. flavus was investigated by the addition of ascorbic acid. (C) H_2_O_2_ content. (D) O_2_^−^ content. (E) CAT activity. (F) POD activity. (G) SOD activity. Values are given as means (*n *= 3) ± standard deviations (*, *P* ≤ 0.05; **, *P* ≤ 0.001).

To confirm the transcriptional analysis results, DEGs linked to oxidative stress and ROS accumulation were verified experimentally. The levels of ROS, H_2_O_2_, and O_2_^−^ and the activities of CAT, POD, and SOD were measured. The results showed that levels of H_2_O_2_ and O_2_^−^ in A. flavus cells treated with estragole were markedly increased compared with those in the control group (Fig. 6A, C, and D). Correspondingly, the addition of 2 mM of the ROS ascorbic acid significantly alleviated the inhibitory effect of estragole on A. flavus at concentrations of 1, 2, and 4 μL/mL (Fig. 6B), and estragole had a strong inhibitory effect on the activities of CAT, POD, and SOD (Fig. 6E to G). These results indicated that estragole might induce the accumulation of ROS in A. flavus cells, which is detrimental to cell growth and metabolism. Additionally, estragole inhibited the activity of antioxidant enzymes and decreased the cells’ ability to scavenge free radicals, resulting in ROS accumulation and elevated levels of H_2_O_2_ and O_2_^−^ in A. flavus.

## DISCUSSION

The essential oil ingredient estragole is increasingly being recognized as safer and more effective than chemical fungicides (27). In this study, we characterized the inhibitory effects of estragole on cell development and aflatoxin synthesis in A. flavus and explored the underlying mechanism by RNA-seq analysis. Additionally, ROS content and antioxidant enzyme (CAT, POD, and SOD) activities were verified experimentally based on transcriptional analysis.

Previous studies indicated that changes in intracellular ROS levels can regulate secondary metabolism (28). When ROS accumulation exceeds the scavenging abilities of antioxidant defense systems, high levels of ROS can lead to oxidative stress and cell damage (29). H_2_O_2_ and O_2_^−^ are by-products of metabolic reactions and major ROS components that play a vital role in regulating growth, development, and secondary metabolism (30). Previous studies have shown that thymol treatment induced ROS production in A. flavus (31), cinnamaldehyde inhibited A. flavus growth and AFB_1_ synthesis by modulating redox status (32), and ROS (including H_2_O_2_) is necessary for apical dominance and growth in Aspergillus nidulans (33). In addition, Zhu et al. (6) demonstrated that deletion of *cta1*, a gene encoding a POD, led to a significant increase in intracellular ROS levels, subsequently increasing oxidative stress levels in cells and ultimately resulting in decreased aflatoxin synthesis. The present study found that following ROS accumulation and increased H_2_O_2_ and O_2_^−^ levels after estragole treatment, growth and aflatoxin synthesis in A. flavus were inhibited, consistent with the results of *afper1* deletion (34). Under these circumstances, cells produce multiple antioxidant enzymes, such as SOD, POD, and CAT, that remove excessive ROS to protect cells from oxidative stress (35). CAT and POD are key enzymes forming the first line of defense against ROS. Both can decompose H_2_O_2_ to produce H_2_O, thereby scavenging H_2_O_2_ (30). In our study, genes encoding antioxidant enzymes (*ctl-2*, *cta1*, *pod*); the glutathione transferase gene *GSTU21*, involved in oxidative stress; and a large number of oxidoreductase-related genes (*2ODD21*, *hxnY*, *yanF*, *yhdF*) were downregulated after estragole treatment, and the activities of SOD, POD, and CAT were significantly reduced. These results indicated that estragole treatment might block AFB_1_ biosynthesis by inhibiting antioxidant enzyme activity and inducing the accumulation of ROS.

ROS accumulation leads to abnormal mitochondrial function and impaired energy metabolism (36). Mitochondria are the site of cellular respiration and energy metabolism, producing ATP through the tricarboxylic acid (TCA) cycle and oxidative phosphorylation. In our study, genes encoding citrate synthase (*r3*, *ACL1*), succinyl coenzyme A (CoA) synthase (*tca-9*), malate dehydrogenase (*mdh*), cis-aconitase (*acoC*), α-ketoglutarate dehydrogenase complex (*kdg1*), and an NADH dehydrogenase component of the respiratory chain complex I (*ndh2*) were downregulated after estragole treatment, similar with the effects of volatile organic compounds produced by Pseudomonas fluorescens against Botrytis cinerea (37). Pyruvate, produced by glycolysis, is located at the intersection of metabolism, and pyruvate dehydrogenase acts as a metabolic switch to convert pyruvate to acetyl-CoA, a vital precursor for aflatoxin biosynthesis (38, 39). We found that glycolytic-related enzyme-encoding genes (*hxkA*, *gld1*, *mug14*), and *PDA1*, encoding pyruvate dehydrogenase, were downregulated, and similar observations were reported following cinnamaldehyde treatment of A. flavus (2). These results suggested that estragole might affect the mitochondrial TCA cycle and other energy metabolic pathways by increasing ROS levels, leading to mitochondrial dysfunction and blockage of metabolic pathways, ultimately inhibiting growth and aflatoxin synthesis in A. flavus.

Accompanied by ROS accumulation and mitochondrial dysfunction, secondary metabolism was disrupted. The aflatoxin biosynthesis pathway requires at least 18 enzymatic reactions and involves up to 30 genes (40). *AflO*, encoding *O*-methyltransferase I, is a key enzyme in aflatoxin biosynthesis (41), and studies have found that loss of *aflO* can significantly inhibit aflatoxin production (42). The *aflU* gene is located at the end of a gene cluster and encodes a polypeptide with a heme-binding domain of cytochrome P450 monooxygenase, involved in aflatoxin biosynthesis (43). A previous study found that the relative expression levels of genes involved in aflatoxin synthesis, such as *aflQ*, *aflO*, *aflP*, and *aflD*, was downregulated after turmeric essential oil treatment (44). *AflT*, which encodes a membrane-binding protein involved in aflatoxin secretion, was highly downregulated by cinnamaldehyde treatment and completely inhibited by citral treatment (45). In our transcriptome data, the aflatoxin biosynthetic pathway genes *aflO*, *aflQ*, *aflU*, *aflT*, *aflB*, and *aflC* were downregulated after estragole treatment. Comparable results were reported for Aspergillus parasiticus treated with curcumin, a Zataria multiflora Boiss. essential oil (46, 47). Additionally, cytochrome P450s, members of the heme-containing monooxygenase superfamily which includes key enzymes in the secondary metabolism of many fungi, can be altered by essential oil treatment (48). Previous studies identified CYP51, a well-studied totipotent enzyme that inhibits ergosterol biosynthesis and causes disruption of cell membranes (49). We found that genes related to cytochrome P450s, such as *dtpC* and *CYP52A2*, were markedly downregulated after estragole treatment, suggesting that estragole disrupts the biosynthesis of cell membrane components and AFB_1_. These results indicated that estragole may inhibit aflatoxin production by regulating multiple genes in the aflatoxin biosynthesis gene cluster.

Estragole treatment also affected the expression of other secondary metabolite synthesis genes. The secondary metabolite ustiloxin B is biosynthesized via the ribosomal peptide synthesis pathway in Ustilaginoidea virens and A. flavus (50, 51). Eighteen genes form a gene cluster for the synthesis of ustiloxin B, and the deletion of certain genes leads to a complete or substantial loss of ustiloxin B production (52). We found that the genes *ustC*, *ustYa*, *ustP*, *ustO*, *ustH*, and *ustD* associated with ustiloxin B synthesis were significantly downregulated. Fungal secondary metabolites are synthesized by gene clusters, which are mostly polyketide synthases (PKSs), nonribosomal peptide synthetases (NRPSs), dimethylallyl tryptophan synthase, or terpene cyclase (53). Polyketides are the most abundant fungal secondary metabolites, and they are synthesized by a type I PKS, a multidomain protein associated with eukaryotic fatty acid synthase (54). Loss of function of *pksCT* in Monascus aurantiacus reduced its ability to produce citrin by more than 98% (55). Nonribosomal peptides are synthesized by multidomain enzymes, specifically nonribosomal polypeptide synthetases, and studies have found that *sid2* is associated with the biosynthesis of siderophores, while *glip* is a widely studied virulence factor involved in gliotoxin biosynthesis (56). Hybrid PKS-NRPS enzymes are involved in the synthesis of mycotoxins such as gibberellin, and of these may be associated with the production of the indoletetranic acid mycotoxin cyclopiazonic acid produced by several species of *Aspergillus* and *Penicillium* (57). In this study, genes associated with PKSs and NRPSs (*pksCT*, *nscA*, *fluP*, *capA*, *aclP*, *NRPS4*) were significantly downregulated. These results indicated that estragole treatment also affected other secondary metabolic pathways, thereby inhibiting the biosynthesis of secondary metabolites.

Estragole treatment also had a significant inhibitory effect on cell growth and development in A. flavus. The expression of genes associated with conidium production in A. flavus is regulated by three transcription factors: *brlA*, *abaA*, and *wetA*. *abaA* is highly expressed in mid-to-late conidia (57) and *wetA* regulates genes associated with conidium formation and metabolism (58). The *stuA* gene acts as a key regulator of fungal development and meristem formation in A. flavus, affecting downstream gene expression by influencing the expression of *brlA* and *abaA*, which in turn leads to the formation of conidia (59). *vosA* is involved in the velvet protein family and functions in the maturation and dormancy of conidia (58, 60). In the present study, four spore-related genes, *wetA*, *abaA*, *vosA*, and *stuA*, were significantly downregulated after treatment with estragole, indicating that estragole might inhibit conidium formation in A. flavus. PI and Hoechst staining results showed that estragole had an inhibitory effect on A. flavus spores, but not a sterilization effect (Fig. S1). Additionally, Boi1 and Bio2 are plasma membrane proteins in budding yeast that are essential for bud growth (61). Cells lacking Boi1 and Bio2 exhibit defective bud emergence and growth and the fusion of vesicles with the plasma membrane (62). Target of rapamycin complexes TORC1 and TORC2 are critical for nutrient utilization (63). TORC1, composed of subunits Tor1, Tor2, Kog1, Lst8, and Tco89, is essential for cell growth and can coordinate various cellular processes and regulate growth (64). Transcriptional analysis showed that growth-related genes such as *boi2* and *kog1* were downregulated after estragole treatment; hence, they may be targets of estragole involved in inhibiting fungal growth. The fungal cell wall, composed of chitin, glucan, and other polymers, is an important structure for the survival and growth of fungal cells (65). Cell wall-related genes were downregulated after estragole treatment. *ags1* and *scw4* are involved in dextran synthesis (66), while *chsD* and *chsE* encode chitin synthase (67, 68). *dcw1* has been shown to be necessary for the incorporation of glycoproteins into the cell wall, and loss of *dcw1* in yeast results in cell wall abnormalities (69). In the present study, after estragole treatment, cell wall-related genes such as *ags1*, *scw4*, *chsD*, *chsE*, *chsA*, *dcw1*, *chit1*, and *gpi13* were downregulated, suggesting that estragole might inhibit mycelium and spore formation in A. flavus, thereby hindering glucan synthesis and causing cell wall damage. Additionally, ergosterol is a vital component of cell membranes, and the *dhcr7* gene encoding C-14 sterol reductase, involved in ergosterol synthesis, was also downregulated, indicating that the biosynthesis of cell membrane components was blocked.

**FIG 7:**
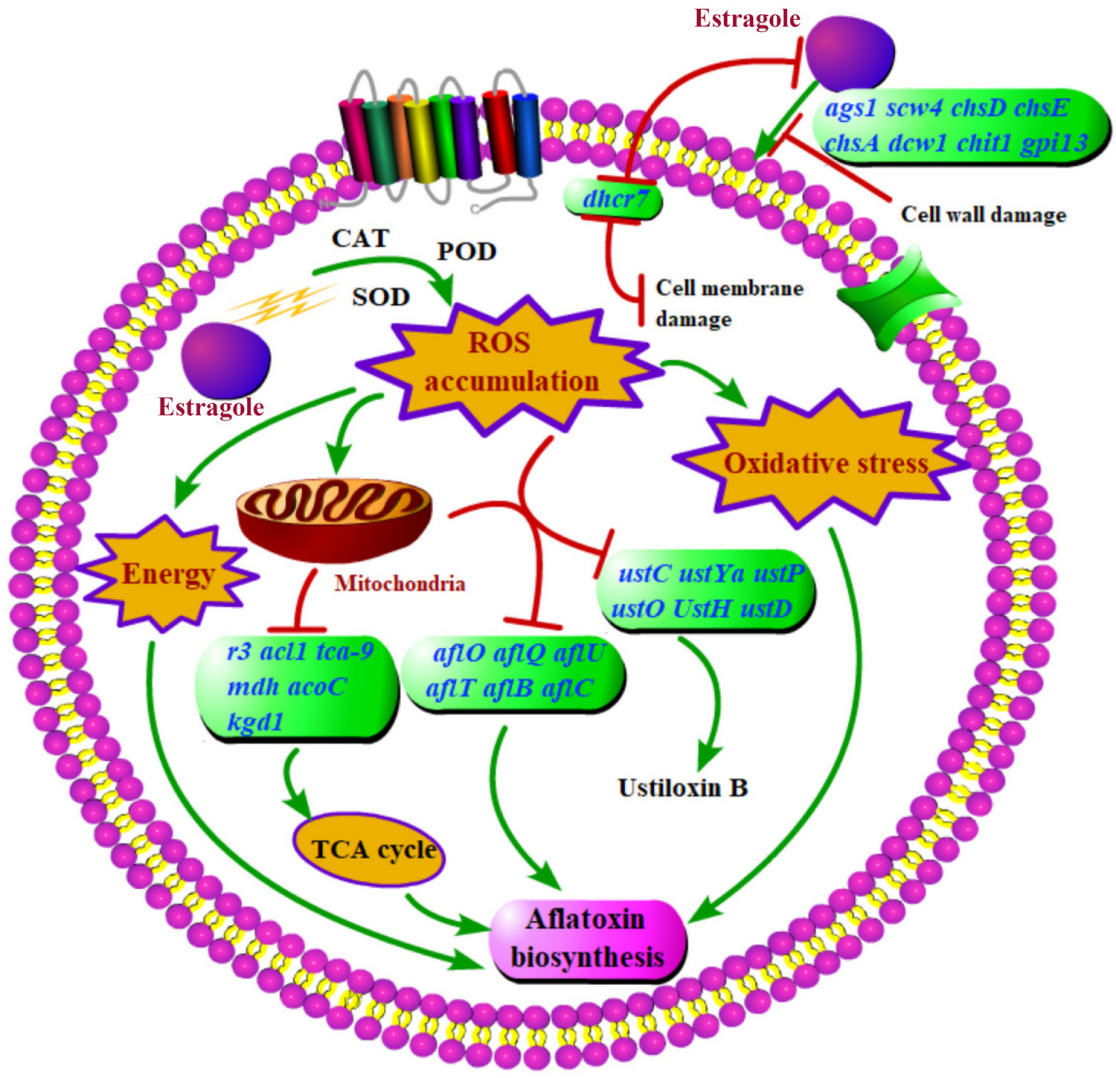
Inhibitory model of estragole on the growth and aflatoxin biosynthesis of A. flavus. Green boxes represent downregulated DEGs in A. flavus treated with estragole compared with the untreated control.

In conclusion, we investigated the effects of estragole on cell growth, development, and aflatoxin production in A. flavus, and explored the underlying mechanism by transcriptome analysis and subsequent verification (Fig. 7). The results indicated that estragole treatment effectively suppressed the growth and aflatoxin synthesis of A. flavus, and effectively inhibited A. flavus contamination in peanut and corn. DEGs were associated with oxidative stress, energy metabolism, and secondary metabolism. Additionally, ROS accumulated, H_2_O_2_ and O_2_^−^ levels increased, and the activities of enzymes involved in antioxidant responses, such as CAT, POD, and SOD, were significantly reduced. These results indicated that estragole inhibited the pathogenicity of A. flavus by modulating intracellular redox homeostasis. These findings broaden our understanding of the antifungal effects and underlying mechanisms of essential oils and illuminate the regulatory mechanism of estragole in aflatoxin synthesis.

## MATERIALS AND METHODS

### Strains and chemicals.

The A. flavus NRRL 3357 strain stored in our laboratory was used in this study. Cells were inoculated on potato dextrose agar (PDA) medium at 30°C for 5 days for spore collection. Estragole was purchased from Aladdin (Shanghai, China). Aflatoxin B_1_ and sterigmatocystin were purchased from Pribolab (Qingdao, China).

### Measurement of the MIC of estragole against *A. flavus* spore germination.

Freshly prepared A. flavus spore suspensions (final concentration: 1 × 10^6^ spores/mL) were inoculated into PDB medium and estragole was added at concentrations of 0.125, 0.25, 0.5, 1.0, and 2.0 μL/mL. After culturing at 30°C for 24 h, A. flavus spore germination was observed by microscopy, and the inhibitory effects of estragole at different concentrations were assessed. The minimum concentration inhibiting spore germination at 24 h was defined as the MIC according to a previous study (70).

### Detection of conidial apoptosis in *A. flavus* due to estragole.

The effects of estragole on the apoptosis of A. flavus conidia were investigated according to previous methods (71). A. flavus spores were treated with estragole at concentrations of 0.5, 1, 2, and 4 μL/mL for 3 and 6 h, conidia were washed once with phosphate-buffered saline (PBS, 10 mM [pH 7.4]), then stained with PI (1 μg/mL) and Hoechst 33258 (10 μg/mL) at 4°C for 30 min in the dark. Apoptotic cells were analyzed by flow cytometry using a CytoFLEX FCM instrument (Beckman Coulter, Indianapolis, IN, USA).

### Effects of estragole on *A. flavus* growth, toxicity, and mycelial dry weight.

The effects of estragole treatment on A. flavus growth were verified by fumigation and solid culture. For fumigation assays, 2 μL of spore suspensions (final concentration 1 × 10^6^ spores/mL) was inoculated on a petri dish containing PDA medium, different concentrations of estragole (0.125, 0.25, and 0.5 μL/mL) were added to the lid, and cultures were sealed at 30°C for 72 h. Colony diameters were measured for each group at 72 h.

Estragole (0.125, 0.25, or 0.5 μL/mL) was added to PDA medium, mixed thoroughly, and poured into plates. After solidification, 2 μL of spore suspensions was inoculated and incubated at 30°C. Colony diameters for each group were measured for 3 days every 24 h, and AFB_1_ content in each group was determined at 72 h. After incubation, the medium was chopped and transferred to a conical flask, an appropriate amount of sterile water and an equal amount of chloroform were added, and the medium was shaken for 30 min at room temperature. After filtering, the chloroform phase was collected, evaporated, and dissolved in 1 mL methanol to obtain AFB_1_. AFB_1_ samples were filtered through a 0.22-μm organic filter and determined by TLC and HPLC. Samples were spotted 1 cm from the bottom of a silica gel plate and placed in a developing solvent of chloroform, acetone (85:15). When the developing solvent had migrated 2/3 of the way up the silica gel plate, it was removed and dried, and the fluorescence intensity of AFB_1_ was examined under UV light at a wavelength of 365 nm. Aflatoxin was also quantified using a Shimadzu LC-2050C 3D instrument with a fluorescence detector and post-column derivatization, and an Agilent ZORBAX SB-C_18_ column (4.6 × 250 mm) with a mobile phase of methanol and water (60:40) at a flow rate of 1 mL/min. The excitation and emission wavelength were 360 nm and 440 nm, respectively. Sterigmatocystin was extracted by chloroform and the content was determined by HPLC with a mobile phase of methanol: water (70:30) at a flow rate of 0.7 mL/min and the UV detection wavelength set at 325 nm.

To investigate the effect of estragole on mycelial weight, PDB medium containing estragole at final concentrations of 0.125, 0.25, 0.5, and 1.0 μL/mL was prepared. After mixing, A. flavus spore suspensions were inoculated and incubated at 30°C with shaking at 200 rpm for 48 h. Supernatants were removed by filtration to obtain mycelia. Mycelia were dried at 80°C for 5 h, and weight was determined according to a previous study (72). AFB_1_ content was determined by the method described above.

### Effects of estragole on growth and aflatoxin biosynthesis in *A. flavus* infecting peanut and corn.

Peanuts and corn were sterilized as described previously (34), and seeds were soaked in A. flavus spore suspensions (10^6^ spores/mL) and shaken for 30 min. Peanut and corn grains were placed into sterile petri dishes with sterile forceps, estragole (0.125, 0.25, 0.5 μL/cm^3^) was added to the lids of petri dishes, and cultures were sealed at 30°C in the dark for 5 days. Infected peanuts and corn were collected into 50-mL centrifuge tubes, 20 mL sterile water was added, and they then were placed in a vibrator for intense vortexing for 2 min. Spore suspensions were collected and conidia were counted by a hemocytometer. AFB_1_ production was extracted and measured according to the methods described above.

### RNA extraction, library construction and sequencing.

Total RNA was extracted from cells using TRIzol (Thermo Fisher, Shanghai, China) according to the manufacturer’s instructions. Briefly, cells were lysed to release RNA, chloroform was added and centrifuged, the upper aqueous layer containing RNA was collected, and RNA was isolated by isopropanol precipitation. An Agilent 2100 Bioanalyzer (Agilent Technologies, Palo Alto, CA, USA) was employed to assess RNA degradation and protein contamination. After total RNA extraction, eukaryotic mRNA was enriched using magnetic beads with oligo(dT). Enriched mRNA was fragmented and reverse-transcribed into cDNA using an NEB Next Ultra RNA Library Prep kit for Illumina (New England Biolabs, MA, USA). The cDNA duplexes were purified, end-repaired, and ligated to Illumina sequencing adapters. Products were purified with AMPure XP microbeads (1×). The sizes of ligation fragments were analyzed by agarose gel electrophoresis and PCR amplification. The resulting cDNA library was sequenced using an Illumina NovaSeq 6000 platform (Gene Denovo Biotechnology Co, Guangzhou, China).

### Bioinformatic analysis.

PCA was conducted using the R software package (http://www.r-project.org/). GO functional analysis was conducted using FungiFun (https://elbe.hki-jena.de/fungifun/help.php). KEGG pathway analysis was conducted using the KEGG Automatic Annotation Sever (KAAS) annotation file.

### Determination of ROS levels, H_2_O_2_, O_2_^−^ accumulation, and the activities of CAT, POD, and SOD.

Determination of ROS levels in A. flavus mycelium using a redox-sensitive fluorescent probe (DCFH-DA) was performed using a ROS detection kit (Beyotime Biotechnology, Shanghai, China). Additionally, the strain was inoculated into PDB medium containing different concentrations of estragole (0.5, 1, 2, and 4 μL/mL), and the ROS scavenger ascorbic acid (0 and 2 mM) was added. The absorbance (optical density at 600 nm, OD_600_) value of the strain was measured by a microplate reader after 36 h of incubation at 30°C (Tecan, Männedorf, Switzerland) to further explore the inhibitory effect on A. flavus. The A. flavus spore suspensions (10^6^ spores/mL) were incubated at 30°C for 24 h, and 0.125 μL estragole was added and incubated for 12 h. After filtering and pressing dry, liquid nitrogen was added and the sample was ground into powder. Levels of H_2_O_2_ and O_2_^−^ in mycelium treated with 0.125, 0.25, and 0.5 μL/mL estragole and in the untreated controls were determined using H_2_O_2_ and O_2_^−^ detection kits (Solarbio, Beijing, China). CAT, POD, and SOD activity after estragole treatment at 0.125, 0.25 and 0.5 μL/mL were also measured using appropriate detection kits (Solarbio).

### Statistical analysis.

All data are represented as means ± standard deviations. Analysis of variance and least significant difference tests were used to compare the significance of differences between two groups, and *P* < 0.05 was considered statistically significant.

### Data availability.

The raw transcriptome read data are available in the SRA database under accession no. PRJNA902091.
