# Supplementary material for: Estragole Inhibits Growth and Aflatoxin Biosynthesis of Aspergillus flavus by Affecting Reactive Oxygen Species Homeostasis
Source: Microbiol Spectr. 2023 Jun 8;11(4):e01348-23. doi: 10.1128/spectrum.01348-23 (PMC10434025; doi:10.1128/spectrum.01348-23)
Supplement: Supplemental file 1 — Supplemental material. Download spectrum.01348-23-s0001.docx, DOCX file, 2.1 MB [file spectrum.01348-23-s0001.docx]

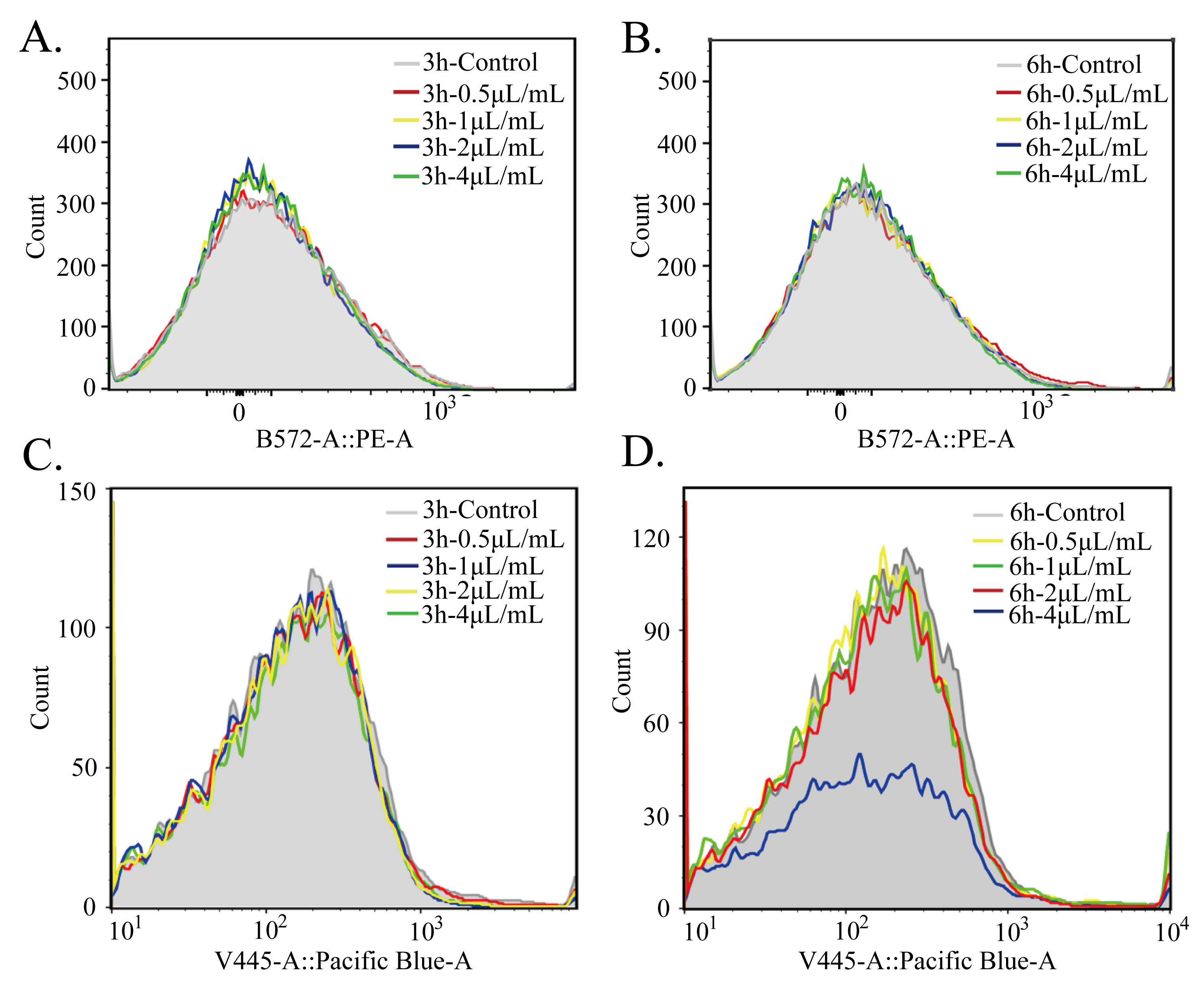


**Fig. S1** Effect of estragole on the spore death of *A. flavus*. (A, B) PI staining of *A. flavus* spores treated with 0.5, 1, 2 and 4 μL/mL estragole at 3 and 6 h, respectively; (C, D) Hoechst staining of *A. flavus* spores treated with 0.5, 1, 2 and 4 μL/mL estragole at 3 and 6 h, respectively.


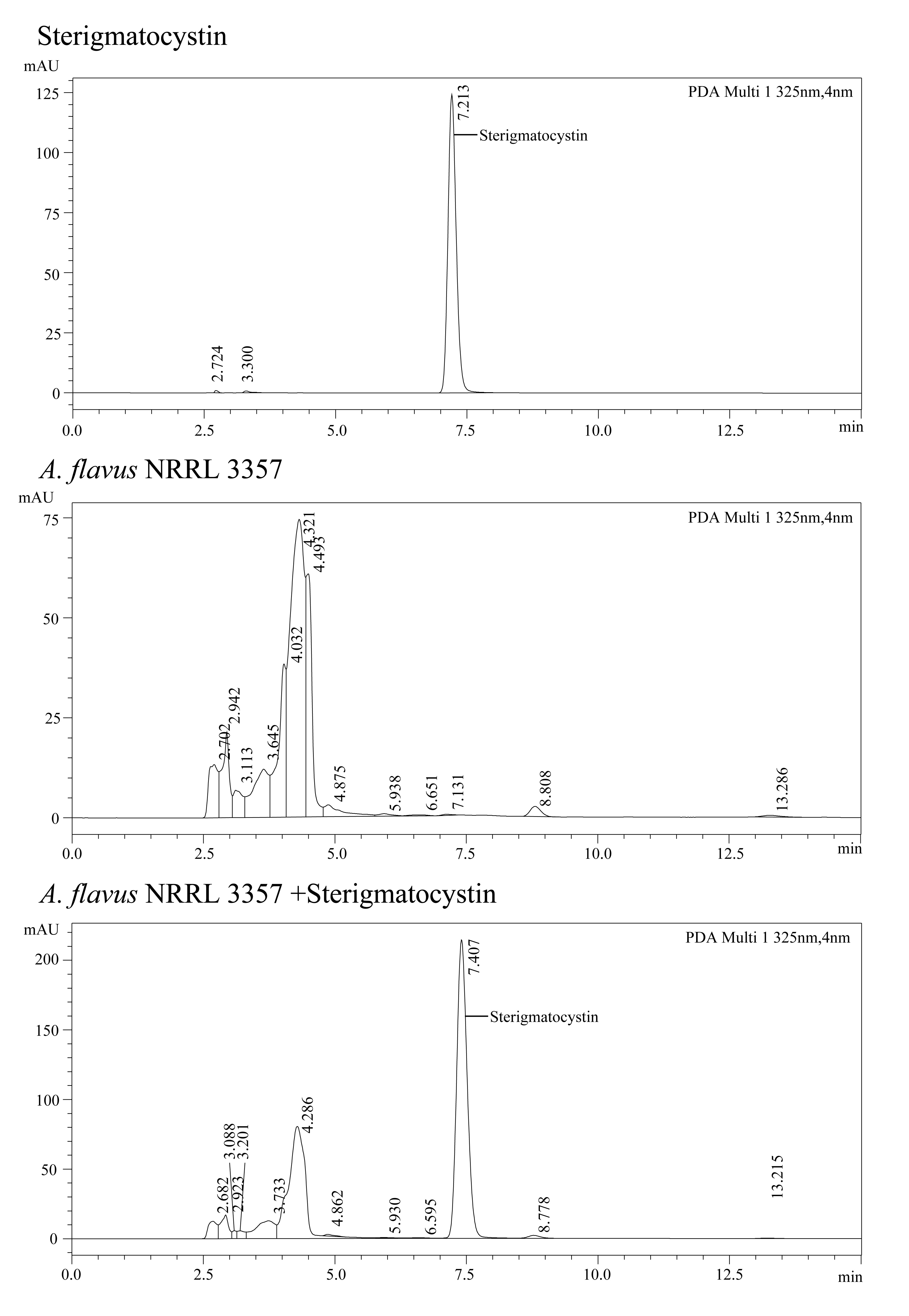


**Fig. S2** HPLC analysis of sterigmatocystin production on PDA medium.
